# Contextualising the WHO Global Research Agenda on Health, Migration and Displacement in Norway invites to a reflection for decolonising research

**DOI:** 10.1186/s12939-025-02410-9

**Published:** 2025-03-04

**Authors:** Esperanza Diaz, Pierina Benavente

**Affiliations:** 1 Department of Global Public Health and Primary Care, University of Bergen, Alrek Helseklynge, Årstadveien 17, 5009 Bergen, Norway; 2 Department for Health and Function, Western Norway University of Applied Sciences, Bergen, Norway

**Keywords:** Health equity, Health research policy, Knowledge management, Migration, Norway

## Abstract

Migrants and displaced persons are ubiquitously present, yet there is insufficient evidence and strategies to provide sustainable, equitable healthcare to these populations globally. Migration and health research has primarily been led by researchers in the Global North (GN), resulting in selective focus that can pose challenges in prioritizing socially relevant questions, and framing migration as a geographically fragmented problem without globally implementable solutions. This power disbalance has recently been termed “colonialisation of research”. The WHO, through an equitable process including the GN and Global South (GS), released the “Global Research Agenda on Health, Migration and Displacement” (Agenda) in 2023 to strengthen globally fair research and translate priorities into policy and practice. WHO invites all countries to contextualise the Agenda´s core research themes and identify national gaps and priorities. With this purpose, the National Research Network for Migration and Health held a workshop in Bergen, Norway, in April 2024. The Norwegian priorities were compared to those from the WHO Agenda and discussed in light of decolonisation of research. Norwegian research priorities align with the WHO Agenda but differ in focus due to national context. Contextualizing the WHO Agenda to specific countries, such as Norway, highlights the need for local relevance while addressing global inequities in research and can, unintentionally, maintain the unresolved challenge of colonialism in research. Future research should critically examine the epistemological and ideological underpinnings of migration and health research to ensure equitable outcomes.

## Background

Migrants and displaced persons exist in most societies, yet we still lack sufficient evidence and strategies to deliver sustainable, equitable healthcare to these populations worldwide. Scientific evidence in the field of migration and health has rapidly developed over the past few decades, but it has primarily been led by researchers in the Global North (GN) with little or no coordination between disciplines and international institutions. Additionally, research has often failed to include the subjects of research or relevant stakeholders as partners [1]. This has contributed to “cherry picking” of the studied diseases and settings, which poses challenges in prioritizing socially relevant research areas, presenting migration as fragmented local problems to be resolved rather than addressing the need for globally implementable solutions.

The acknowledgment of colonialism in education and research has gained importance in the last decade [2]. Norway, as one of the richest countries in the GN has also been part and parcel of research colonialism, but is slowly starting to acknowledge its role in this complex challenge [3]. Decolonising science is a critical movement aimed at challenging and transforming the GN´s biases and power dynamics embedded in science production. Related to research on global health, Kumar et al. present colonialism in three dimensions: *colonisation within global health research*, related to who leads research; *colonisation of global health research,* or who controls the agenda of global health research; and *colonisation through global health research,* related to who benefits from such research [4].

Formed through an inclusive, equitable process including both the GN and the Global South (GS), the WHO released a technical document entitled “Global Research Agenda on Health, Migration and Displacement” in 2023 to advance knowledge production and implementation. Through this effort, the WHO sets out its first global research priorities in this area. The Agenda aims to strengthen research and translate research priorities into policy and practice, and to support national and regional efforts to address the health needs of migrants and displaced populations within the framework of the Sustainable Development Goals.

The Agenda identifies key challenges in five main areas organised into three core research themes (i-iii) and two cross-cutting research themes (iv, v): (i) scaling up access to services and universal health coverage (UHC), (ii) making health emergency preparedness more responsive, (iii) better understanding determinants of health, (iv) bringing visibility to under-researched groups, and (v) finding new ways to collaborate in research. Six research subthemes under the three core themes were further prioritized (Table 1).

**Table 1:** WHO research subthemes under the three core themes in the Global research Agenda [6]

**Core theme 1: **generate evidence on inclusive UHC and primary health care (PHC) for migrants, refugees and other displaced populations (MRD): **Priority subtheme 1.1 **– effective models of health financing for MRD **Priority subtheme 1.2 **– interventions to improve the responsiveness of service provision to diversity
**Core theme 2: **improve knowledge generation on the inclusion of MRD in preparedness and response to (health) emergencies: **Priority subtheme 2.1 **– effective and sustainable models of health care for MRD in humanitarian settings in low- and middle-income countries (LMICs) and fragile contexts **Priority subtheme 2.2 **– effective models of UHC in protracted displacement contexts
**Core theme 3: **generate multisector research on addressing the determinants of health of MRD: **Priority subtheme 3.1 **– impact of living and working conditions on the health of MRD **Priority subtheme 3.2 **– impact of restrictive immigration policies, securitization and borders on the health of MRD

Since sociopolitical contexts differ across continents and countries, future research on these areas requires regional and national contextualization. Also, the development of specific research questions for each subtheme, tailored to the needs of each setting, is encouraged by WHO. However, the research contexts also differ, with the GN usually having more resources both for the exercise of contextualisation and for research itself, which could, unintentionally, perpetuate colonialisation of research.

The National Research Network for Migration and Health (NFMH) is an open network encompassing researchers in migration and health in Norway [5]. The network includes researchers in diverse disciplines interested in the research field. Established in 2019 and led by the University of Bergen, NFMH’s goal is to generate better and more robust knowledge to illuminate and enhance the health of and healthcare services for migrants in Norway. To align NFMH’s priorities with those of WHO and enhance general networking among members, the network organized a participatory exercise to contextualise WHO’s themes and subthemes to Norway in April 2024, in Bergen, Norway. Fourteen NFMH members (10% of the network) attended the meeting in-person, including one research assistant, two master students, five PhD candidates, three post-doctoral fellows and three senior researchers from different academic institutions in Norway.

To better adapt to the Norwegian context, which has no displaced populations and where most migrants are labor migrants, we used the term “migrant” as defined in the Agenda: “A person who moves permanently or temporarily from their usual place of residence to another, whether across or within international boundaries” instead of the Agenda’s “migrants, refugees and other displaced populations (MRD)” [6]. A digital presentation of the Agenda was delivered by a WHO representative, including the thorough presentation of the core themes. The audience was thereafter divided into three groups, each group focusing on one of the core research themes of the Agenda. The participants joined the group most related to their field of interest. A representative from the University of Bergen was assigned to each group to take notes and facilitate the discussion process. Each group was provided with a printed list of the WHO ranked research priorities for their chosen theme and invited to address two questions: “What are the gaps for inclusiveness in Norway?” and “What are the priorities in Norway, comparing the Global Research Agenda's priorities with those in Norway?”.

Each group’s responses were shared in a plenary session, followed by an open discussion of the two cross-cutting themes in light of the responses to the core themes. Notes were taken on a whiteboard after each comment, asking for confirmation to ensure an accurate understanding of participants’ contributions. The plenary discussion was thereafter summarized by the first author with all members present, and this information, along with the written notes from each group, was used to draft the document outlining gaps and priorities for Norway.

### Prioritised gaps in research in Norway

The gaps in research identified for Norway during the workshop overlapped and were therefore further re-grouped by the paper’s authors into four domains: (i) methodology, (ii) interdisciplinarity and co-production of research, (iii) implementation of evidence and (iv) specific knowledge gaps.

### Methodology

Several methodological needs were highlighted, including the need to:

- work towards a coherent use of research terms across researchers and disciplines (coherence)
- collect more comprehensive and disaggregated data to distinguish between various migrant groups (granularity)
- incorporate robust variables and indicators of migrant background in regular health surveillance and existing data panels (mainstreaming)
- increase data collection on determinants of health, such as sexuality, socioeconomic status and migrant background (other determinants of health)
- simplify and make more easily available access to linked data for research including migration and socioeconomic variables (linkage)
- develop and use validated tools and research instruments for migrant groups (validation)
- facilitate intervention studies

### Interdisciplinarity and co-production of research

Interdisciplinarity and co-production were seen as two distinct but necessary cornerstones for research with a commonality of challenging dialogues between complementary parts. The gaps and needs identified in this field were:

- There are administrative and theoretical barriers to collaboration between disciplines and sectors despite a general understanding that health is impacted by policies in all areas of knowledge.
- A need to work systematically to create and maintain trust and to share existing knowledge with migrant communities and organizations as keys to high quality research.
- A need to facilitate participatory approaches to research, planning and dissemination, for example by creating reference groups with members from different disciplines and institutions for research projects.

### Implementation of research

As is the case in other research fields, the discrepancy between knowledge and action is a major challenge regarding migration and health, but the situation is further aggravated by migration being politically controversial. In many cases, evidence exists at the national level to support more equitable healthcare, but there is no evidence or political will to implement the necessary changes. Some examples named were the unresolved needs of:

- Increasing cultural competence among healthcare professionals.
- Improving health literacy among migrants.
- Providing translated information.
- Engaging with key individuals working with vulnerable groups.

### Specific knowledge gaps

In addition to gaps in knowledge regarding implementation, the following themes were identified as lacking evidence in Norway:

- Discrimination and health, social integration and health and health promotion.
- Adaptability of health systems to meet the needs of those seeking care.
- Migrants' healthcare needs, quality of the available services provided and venues for improvement.
- Culturally sensitive preventive care.
- Oral health.

### Prioritised subthemes in Norway considering the WHO Research Agenda

Finally, the Norwegian priorities (Table 2) were compared to those in the WHO research agenda. As previously explained, Norwegian priorities focused on international migrants and did not include displaced or receiving populations or LMIC settings. Although two of the top three Norwegian priorities were the same as in the WHO Agenda, in several cases lower-ranked Norwegian priorities were much further down on WHO’s list. Furthermore, particular methodological or thematic problems were specified in Norway for the shared priorities, making the focus of the subthemes somehow different. For instance, under the subtheme “Development of sustainable models of transition for better healthcare for migrants in Norway”, our participants specified the need to study how to change from a non-migrant-sensitive to migrant-sensitive health care model and incorporate cultural competence and safety into the system. Other research areas that should be specifically targeted in Norway included focus on trust and representation in research, facilitating healthcare access for undocumented migrants, and addressing discrimination as a main sociopolitical factor in society affecting health. Conducting research related to the implementation of evidence was highlighted as key to addressing the existing gaps in determinants of health in Norway not only within the particular “gaps in implementation of research” theme but also as a cross-cutting theme.

**Table 2:** Research priorities in Norway under the three core themes

**Core theme 1: Inclusive UHC and PHC for migrants**
Priority subtheme 1.1: Effective models of health financing to better include migrant populations Priority subtheme 1.2: Good practices in resource allocation for achieving equity in health entitlements Priority subtheme 1.3: Means by which migrants care for themselves Priority subtheme 1.4: Cross-sectoral approaches and solutions facilitating healthcare access, including those based on the right to health
**Core theme 2: Inclusion of MRD in preparedness and response to (health) emergencies**
Priority subtheme 2.1: Development of sustainable models of transition for better healthcare for migrants in Norway Priority subtheme 2.2: Health needs of migrants prior to crisis and including them in preparedness plans Priority subtheme 2.3: Impact of sociopolitical factors on the delivery of health services Priority subtheme 2.4: Analysis of modalities of health outcomes in the implementation of policies that include migrants
**Core theme 3: Multisectoral research on addressing the determinants of health of migrants**
Priority subtheme 3.1: Understanding the effect of discrimination and marginalization on health and well-being Priority subtheme 3.2: Impact of language/cultural background on health Priority subtheme 3.3: Researching and improving governance of migration health Priority subtheme 3.4: Research on strengthening capacity building initiatives Priority subtheme 3.5: Interdisciplinary and transdisciplinary approaches in research Priority subtheme 3.6: Conducting implementation research to bridge policy and practice

Several under-researched groups were highlighted in Norway, related to different axes, like migrant status (including undocumented migrants and specific groups of refugees and labour migrants), life trajectory (children, adolescents, elderly) and generations (children of migrants), religious minorities, and migrants with low educational status. Gender differences and intersectionality, or the crossing interaction of belonging to several groups, were mentioned as important research lenses, rather than studying the groups in silos. These groups were somehow different from those presented in theme 4 in the WHO Agenda.

Regarding how to strengthen equitable and inclusive research collaboration and knowledge translation into policy and practice described as theme 5 in the WHO Research Agenda, the priorities in Norway were to focus on using validated tools or validating research instruments for migrant groups, relying on participatory approaches to research, planning and dissemination and creating reference groups for research projects with members from different disciplines and institutions. The participants also suggested that researchers should make special efforts to provide actionable evidence for policy and practice by understanding how policymakers make decisions and presenting actionable results linked to specific challenges.

## A reflection for decolonising research

The WHO´s agreement on a Global Research Agenda is a welcome effort to establish global priorities that can contribute to research helping society understand and improve the health of groups in vulnerable situations and, in the long term, view migration as a driver of health.

Our results regarding research priorities in Norway, using the WHO framework, align with previous papers and declarations in Norway over the past years, although with minor discrepancies [7–11].

Norway´s priorities identified in this paper also generally align with those presented by the WHO Agenda, but with some noteworthy deviations in terms of subjects and areas of interest. The first significant difference is the focus on which subgroups of migrants should be the subject of study. Definitions and terminology are important for any research field, and to a certain degree can direct the research conducted towards specific areas of interest. In Norwegian statistics, migrant background is typically categorized by country of origin. Thus, the term migrant is used in a broad sense, including refugees, labour migrants, family reunification migrants and educational migrants. Most migrants, regardless of their reason for migration, live integrated in municipalities, and there are no internally displaced populations in the country. In this context, Norwegian researchers did not prioritise displaced populations, vulnerable receiving populations or settings in LMICs. Moreover, all legally residing migrants in Norway are entitled to healthcare services, except for undocumented migrants who have limited rights. Therefore, the undocumented group was often mentioned even though there is growing research in this particular topic [12–17].

Another notable difference, also likely due to national context and not to differences in the research perspective, concerns research settings. WHO’s priority subthemes anchored in low resource settings and fragile contexts (2.1 in Table 1); in protracted displacement contexts (2.2 in Table 1); or regarding restrictive immigration policies, securitization and of borders on the health of MRD (3.2 in Table 1) were not considered relevant to Norway. On the other hand, themes such as trust and representativeness, which were very relevant for Norway´s dissemination of information during the pandemic [18–21], and discrimination, are unfortunately increasingly challenging in Norwegian and European settings [22–24] and were acknowledged as main research priorities. Next, implementation research on capacity building, including cultural competence, safety and health literacy aimed at enhancing the quality and equity of care was higher prioritised in Norway as compared to the WHO Agenda, and these subjects were categorized as specific subthemes within the multisectoral approach needed to address other determinants of health, aligning with earlier publications [25, 26].

Considering these reflections, we believe the gaps and priorities identified in Norway could be similar in other Nordic countries, and perhaps in other GN countries with comparable migrant populations, health care services and health care entitlements for migrants in Northen Europe. However, while anchoring the agenda locally is recommended by the WHO, this effort might unintentionally maintain the unresolved challenge of colonialism in research by giving priority in the GN, with more research resources, to challenges and groups that are not the most precarious in the GS. This in turn would increase the gap in research given the inability of countries of the GS to weigh in on the migration and health research agenda because of historical and persisting inequity-generating mechanisms. This possibility poses dilemmas that merit further reflections.

Following the principle of conducting socially relevant research at the local level and aiming for research led by the very same groups that are the subject of study, research should be grounded in the populations and contexts of each country, including those in the GN [2]. By this logic, it would be challenging -or even inappropriate- for researchers working in the Norwegian context to focus for example on internally displaced populations or on the lack of primary health care entitlements, as these are not part of their contextual realities. Thus, contextualizing the Agenda at the national level, as proposed by the WHO, could unintentionally contribute to maintaining the status quo by focusing on areas of research that are not prioritised in the GS. Besides, leaning on Kumar et al.´s dimensions of colonialism [4], there is no doubt that research in the GN has better academic and economic support and infrastructure compared to the GS. This fact can further strengthen colonisation *within* (lead) and *of* (control) migration and health research, raising the question of to what degree researchers in Norway should compromise their own priorities and put further effort to collaborate with countries in the GS, in order to decolonise research and achieve equity in *who* benefits from such research [4]. Without straightforward answers that fit all research projects, these dilemmas call for a critical examination of the epistemological and ideological underpinnings of setting priorities in research in migration and health research that we believe should be clearly addressed in the future development of the Global Research Agenda.

We acknowledge that our contextualization and prioritization of the WHO Agenda has several limitations. Despite efforts to include as many Norwegian researchers in the field of migration and health as possible, the participants in the workshop represented only ten percent of those in our network, which itself does not encompass all researchers in the field. We decided to organize the workshop on site to enhance networking among Norwegian members. However, this choice might partially explain the low turnout for the workshop, as the need for own funding for travel and accommodation hindered participation for some researchers. For similar activities in the future, allowing on-line presence might enhance participation. Regarding the methods used to agree on the priorities, the WHO Agenda includes a Toolkit to adapt and expand upon the agenda at regional, national and local levels that we chose not to follow due to its complexity. Although engaging several stakeholders in a more complex process, as suggested by the Toolkit, might have yielded different results, such a process was not feasible in practice.

## Conclusion

Contextualising the WHO Global Research Agenda is a necessary effort to advance the research field of migration and health in a realistic, coordinated way that includes local stakeholders in a common effort to improve health for all populations. However, the task is complex and requires self-reflection to continue decolonialising research.

## Data Availability

Data is provided within the manuscript
